# Emerging Role of Mesenchymal Stromal Cell and Exosome Therapies in Treating Cognitive Impairment

**DOI:** 10.3390/pharmaceutics17030284

**Published:** 2025-02-20

**Authors:** Vick Key Tew, Muttiah Barathan, Fazlina Nordin, Jia Xian Law, Min Hwei Ng

**Affiliations:** Department of Tissue Engineering and Regenerative Medicine, Faculty of Medicine, Universiti Kebangsaan Malaysia, Cheras, Kuala Lumpur 56000, Malaysianordinf@ppukm.ukm.edu.my (F.N.); lawjx@ppukm.ukm.edu.my (J.X.L.)

**Keywords:** cognitive aging, mesenchymal stromal cell, exosome, cognitive impairment, regenerative medicine

## Abstract

Cognitive aging, characterized by the gradual decline in cognitive functions such as memory, attention, and problem-solving, significantly impacts daily life. This decline is often accelerated by neurodegenerative diseases, particularly Alzheimer’s Disease (AD) and Parkinson’s Disease (PD). AD is marked by the accumulation of amyloid-beta plaques and tau tangles, whereas PD involves the degeneration of dopaminergic neurons. Both conditions lead to severe cognitive impairment, greatly diminishing the quality of life for affected individuals. Recent advancements in regenerative medicine have highlighted mesenchymal stromal cells (MSCs) and their derived exosomes as promising therapeutic options. MSCs possess regenerative, neuroprotective, and immunomodulatory properties, which can promote neurogenesis, reduce inflammation, and support neuronal health. Exosomes, nanosized vesicles derived from MSCs, provide an efficient means for delivering bioactive molecules across the blood–brain barrier, targeting the underlying pathologies of AD and PD. While these therapies hold great promise, challenges such as variability in MSC sources, optimal dosing, and effective delivery methods need to be addressed for clinical application. The development of robust protocols, along with rigorous clinical trials, is crucial for validating the safety and efficacy of MSC and exosome therapies. Future research should focus on overcoming these barriers, optimizing treatment strategies, and exploring the integration of MSC and exosome therapies with lifestyle interventions. By addressing these challenges, MSC- and exosome-based therapies could offer transformative solutions for improving outcomes and enhancing the quality of life for individuals affected by cognitive aging and neurodegenerative diseases.

## 1. Introduction

Cognitive aging is the natural process of changes in thinking, learning, and memory that occur as people age, involving a mix of decline, stability, or growth across different cognitive domains [1]. While some areas, such as processing speed, sustained attention, multitasking, working memory, and word retrieval, may show decline, others may remain stable or even improve with age [2]. This process is influenced by various factors, including lifestyle, sleep, diet, and physical activity [3]. Adopting healthy habits such as getting adequate sleep, eating nutritious foods, staying physically active, engaging in social activities, and keeping the mind stimulated can help mitigate cognitive decline and support healthy cognitive aging [4]. Another study also mentioned that lifestyle factors such as higher education, moderate alcohol consumption, and physical fitness contribute significantly to cognitive resilience [5]. Neurophysiologically, aging is associated with structural brain changes, such as reduced volume and altered functional activation patterns, which may serve as compensatory mechanisms to counteract decline [6]. Cognitive control, crucial for adaptability and multitasking, also changes with age, influencing daily life and work performance [7].

Cognitive impairment, in contrast, refers to clinically significant deficits in cognitive function that interfere with daily life [8]. It ranges from reversible conditions, like delirium caused by infections or medication toxicity, to progressive and irreversible disorders like dementia, including Alzheimer’s Disease (AD), Parkinson’s Disease Dementia (PDD), and vascular dementia [9]. Causes of cognitive impairments include neurodegenerative diseases, cerebrovascular conditions, and systemic factors such as diabetes and nutritional deficiencies [10]. Psychiatric conditions like depression, as well as lifestyle factors like smoking and inactivity, further exacerbate cognitive decline [11].

As life expectancy increases, maintaining cognitive function and quality of life becomes a critical societal challenge. In this context, mesenchymal stromal cells (MSCs) and their exosomes are emerging as promising therapeutic options for managing cognitive impairment and neurodegenerative diseases [12]. MSCs possess regenerative and immunomodulatory properties that help reduce inflammation and neuronal damage in conditions like AD and Parkinson’s Disease (PD) [13]. MSC-derived exosomes, containing bioactive molecules, offer a novel approach to slowing disease progression and alleviating symptoms [14]. Administered early, they have the potential to improve outcomes for individuals at risk of cognitive decline.

This review focuses on cognitive aging and cognitive impairment associated with AD and PD, two prominent neurodegenerative conditions. Both AD and PD exemplify the continuum between normal cognitive aging and pathological cognitive decline, sharing overlapping mechanisms such as neuroinflammation, oxidative stress, and structural brain changes. AD is primarily associated with progressive memory loss and executive dysfunction, driven by amyloid-beta plaques and tau pathology. In contrast, PD-related cognitive impairment often presents with deficits in attention, executive function, and visuospatial abilities, linked to dopaminergic neurodegeneration and alpha-synuclein pathology. By examining these conditions, this review aims to elucidate the interplay between typical age-related cognitive changes and disease-specific impairments, offering insights into shared and distinct mechanisms and potential avenues for intervention. It also highlights the multifactorial nature of cognitive decline and the therapeutic potential of MSCs and MSC-derived exosomes in managing cognitive impairment and neurodegeneration. These emerging therapies offer hope for improving patient outcomes and quality of life.

## 2. Methodology

A comprehensive literature search was conducted to gather relevant studies and reviews on cognitive aging, cognitive impairment, AD, PD, and the therapeutic potential of MSCs and their derived exosomes. The search was performed using four major databases: PubMed, Scopus, and Google Scholar. Keywords such as “cognitive aging”, “cognitive impairment”, “Alzheimer’s Disease”, “Parkinson’s Disease”, “mesenchymal stromal cells”, and “exosomes” were used in combination with Boolean operators to refine the search results. Studies published in English within the past 10 years were included to capture the most up-to-date findings. This approach ensured a thorough review of the literature, covering both fundamental research on neurodegenerative diseases and emerging therapeutic strategies involving MSC- and exosome-based treatments. By focusing on recent advancements, the search aimed to highlight current trends and gaps in the field, thereby laying the foundation for a discussion of the therapeutic potential of MSCs and exosomes in addressing cognitive decline and neurodegenerative diseases.

## 3. Cognitive Aging Versus Cognitive Impairment

Cognitive aging typically begins in early adulthood, with subtle declines in memory and reasoning becoming more noticeable as individuals grow older [15]. It is a natural and non-pathological process characterized by gradual reductions in processing speed, memory, attention, and executive functions [16]. However, some abilities, such as vocabulary and problem-solving using accumulated knowledge, often remain stable or even improve into advanced age [17]. Unlike dementia, cognitive aging reflects structural changes in the brain and nervous system rather than disease, significantly impacting cognitive function. Brain structural aging exhibits significant inter-individual variability, with some individuals showing accelerated patterns linked to cognitive decline and increased susceptibility to neuropsychiatric disorders [18]. These variations highlight the importance of identifying risk factors and potential biomarkers to predict cognitive outcomes [19]. Cognitive aging is characterized by structural changes in key brain regions, including the prefrontal cortex (PFC), thalamus, hippocampus, and cortical sulci, which significantly impact cognitive functions such as memory, executive function, and fluid intelligence [20]. The PFC shows increased structure–function coupling associated with declines in executive function, while thalamic changes are linked to reduced fluid intelligence [21]. The hippocampus exhibits molecular alterations and synaptic loss, particularly in males, contributing to memory impairments, whereas atrophy in the tertiary sulci of the posteromedial cortex correlates with deficits in memory and executive functions [22]. In addition, the integrity of white matter is crucial for efficient neural communication and cognitive performance. Declines in white matter integrity are associated with poorer cognitive outcomes, whereas its preservation is a hallmark of successful cognitive aging [23]. Despite structural changes, some individuals demonstrate cognitive resilience through adaptive mechanisms, highlighting the brain’s plasticity and capacity for compensation. For instance, increased reliance on alternative neural pathways or enhanced connectivity in unaffected regions can help maintain cognitive function, even in the face of structural decline [24]. The pace of cognitive aging varies across individuals due to genetic and lifestyle factors. While genetics influence resilience, factors such as a healthy diet, regular exercise, and social engagement are associated with slower decline [25]. Chronic conditions like hypertension and diabetes can exacerbate cognitive aging through vascular and inflammatory mechanisms. Mitigating cognitive aging involves strategies like cognitive training, lifestyle changes, and stress management [26]. Exercises targeting memory, attention, and executive function are effective, while social interaction and lifelong learning help maintain cognitive health. These approaches promote healthy aging and reduce the risk of significant cognitive decline.

In contrast, cognitive impairment involves a significant decline in cognitive function that interferes with daily life, distinct from normal aging [27]. It may manifest as memory loss, difficulties in communication, problem-solving, or judgment. Cognitive impairment can result from a range of neurological, medical, and systemic conditions. Neurological disorders such as AD, PD, vascular dementia, and traumatic brain injury are prominent contributors [28,29]. Medical conditions, including stroke, diabetes, and chronic obstructive pulmonary disease (COPD), are also significant factors [30]. Cerebral small vessel disease has been shown to exacerbate cognitive decline in AD and PD [31]. Autism spectrum disorder, chemotherapy-induced cognitive impairment, and schizophrenia exhibit overlapping deficits in memory and executive function, often paralleling neurodegeneration [32]. Emerging evidence links COVID-19 to cognitive decline, likely through mechanisms involving inflammation and oxidative stress [33]. Additionally, obesity-related cognitive impairment, associated with systemic inflammation and gut microbiome dysregulation, has been implicated in AD pathology [34]. These diverse causes highlight the complex interplay of neurological, metabolic, and systemic factors in cognitive impairment. Diagnosis involves a combination of neuropsychological assessments, laboratory tests, and medical evaluations. Tools like the Mini-Mental State Examination (MMSE) and the Montreal Cognitive Assessment (MoCA) are commonly used to evaluate cognitive function and identify the extent of impairment [35]. Blood tests check for reversible factors such as vitamin deficiencies or metabolic issues, while imaging techniques like MRI or PET scans detect brain atrophy, lesions, or amyloid plaques [36]. Additional evaluations may include cerebrospinal fluid analysis for biomarkers, EEG for abnormal brain activity, and psychiatric screening to rule out depression or anxiety [37]. This multifaceted approach ensures accurate identification and management of cognitive decline.

Table 1 displays the key differences and similarities between cognitive aging and cognitive impairment.

## 4. The Link Between AD and PD with Cognitive Aging and Impairment

AD and PD are intricately linked to cognitive aging through both shared and distinct neurobiological mechanisms [38]. Aging is a significant risk factor for both conditions, driving cognitive decline through processes such as mitochondrial dysfunction, chronic inflammation, and cellular senescence, which impair neuronal function and exacerbate neurodegeneration [39,40]. These shared mechanisms create a common foundation for age-related cognitive impairment in AD and PD, yet their clinical manifestations and pathological trajectories remain distinct. AD is characterized primarily by episodic memory deficits due to hippocampal atrophy, amyloid-β plaques, and tau tangles [41], whereas PD predominantly affects visuospatial abilities, attention, and executive function, driven by dopaminergic neuronal loss and disruptions in frontal-striatal circuits [42]. Brain network alterations further differentiate the two diseases, with AD exhibiting less segregated resting-state networks indicative of widespread synaptic dysfunction, while PD shows distinct network disruptions in dopaminergic pathways [43]. Despite these differences, both conditions exhibit some convergence in advanced stages, with overlapping deficits in attention, language, and executive function reflecting shared downstream neurodegenerative processes [44]. This interplay between shared biological aging mechanisms and disease-specific pathologies underscores the complexity of cognitive decline in aging, emphasizing the need for targeted interventions that address both common and unique aspects of AD and PD.

Meanwhile, the relationship between AD and PD with cognitive impairment is both intricate and multifaceted, as these neurodegenerative conditions exhibit distinct yet overlapping cognitive deficits that evolve differently over time [45]. Cognitive impairment is a hallmark of both diseases, with mild cognitive impairment (MCI) often presenting early in PD, while AD is predominantly associated with progressive memory loss and dementia [46]. In PD, cognitive impairment is prevalent, affecting up to 75–90% of patients as the disease progresses to dementia. The deficits are commonly characterized by dysexecutive syndrome and visuospatial disturbances, profoundly impacting patients’ quality of life [47,48]. Neuroimaging studies have linked these impairments to white matter abnormalities and cortical thinning, underscoring the neuroanatomical disruptions associated with PD. In contrast, AD-related cognitive impairment (ADCI) primarily manifests as memory dysfunction, with notable deficits in attention and executive function, driven by pathologies in the medial temporal region and widespread cortical thinning [49]. While both conditions share some neurodegenerative processes, such as mitochondrial dysfunction and inflammation, their underlying mechanisms diverge, with AD showing pronounced cortical atrophy and PD associated with dopaminergic system disruptions and white matter change [50,51]. These distinctions are reflected in their neuropsychological profiles and brain network alterations, emphasizing the need for tailored therapeutic interventions. Understanding the shared and unique cognitive and pathological features of AD and PD is crucial for advancing research, optimizing treatment strategies, and improving outcomes for individuals affected by these diseases.

## 5. Pathogenesis of AD and PD

AD, the primary type of dementia (making up 60–80% of cases), is also the primary reason for dementia among the older population worldwide [52]. AD is defined by a gradual decline in neurological function that impairs cognitive abilities and memory, making it difficult to carry out daily tasks [53]. Symptoms typically start with mild memory loss and progress to severe cognitive decline. While younger individuals can also be affected, it is more common in those over 65. The number of affected individuals is projected to nearly triple to 14 million by 2060 [54]. This growing occurrence places a rising financial strain on people, families, and the community as a whole. AD is characterized by significant brain atrophy due to the loss and malfunctioning of neurons. This process leads to the breakdown of neuronal networks and shrinkage of brain regions, particularly in the final stages of the disease [55]. The hippocampus and entorhinal cortex, crucial for memory formation, are among the first areas to suffer damage. As the disease progresses, other brain regions shrink, including the temporal and parietal lobes, parts of the frontal cortex, and the cingulate gyrus, resulting in a noticeable loss of gyri and sulci [56]. The rate of brain atrophy in AD is significantly accelerated, occurring two to ten times faster than in individuals of the same age without the disease [57].

The PI3K/Akt/mTOR signaling pathway plays a crucial role in various cellular functions and is significantly disrupted in AD. Research indicates that the abnormal activation of this pathway occurs early in AD [58]. This hyperactivation results in dysfunctional autophagy, increased production of amyloid-beta (Aβ), and hyperphosphorylation of tau proteins, ultimately contributing to synaptic dysfunction from amyloid oligomers, disrupted neuronal transport from tau tangles, neuroinflammation, oxidative stress, and mitochondrial dysfunction, ultimately resulting in extensive neuronal death, particularly in memory/cognitive areas such as the hippocampus [59,60]. Genetic factors such as apolipoprotein E gene called *APOE4* increase the risk of AD by affecting the clearance of amyloid [61]. The mitochondrial cascade hypothesis suggests that alterations to mitochondrial DNA (mtDNA) and nuclear genes affecting the electron transport chain in AD impact mitochondrial function, leading to higher levels of ROS, mtDNA damage, reduced energy production, and oxidative stress [62].

PD is the second most common neurodegenerative disorder after AD, known for its progressive motor symptoms like tremors, rigidity, bradykinesia, and postural instability, but as it progresses, many patients also develop cognitive impairments [63]. In the past few years, PD has experienced a fast rise in occurrence and disability, emerging as a top reason for disability on a global scale [64]. PD is a progressive neurodegenerative disorder primarily characterized by the loss of dopaminergic neurons in the substantia nigra pars compacta, a critical region of the brain involved in controlling movement [65]. This neuronal loss leads to hallmark motor symptoms such as tremors, rigidity, bradykinesia (slowness of movement), and postural instability. The progressive nature of PD results in increasing difficulty with voluntary movements, severely impacting the quality of life for affected individuals [66]. The pathogenesis of PD involves a complex interplay of genetic, environmental, and biochemical factors. Inflammation and oxidative stress are central to the disease’s development. NADPH oxidases, enzymes that produce ROS, contribute to neuroinflammation and oxidative damage, which in turn exacerbate neuronal injury and dysfunction [67]. These oxidative processes are closely linked to the accumulation of alpha-synuclein, a protein that forms toxic aggregates known as Lewy bodies, a pathological hallmark of PD [68]. Mitochondrial dysfunction is also a significant factor, as impaired energy production in neurons can lead to increased oxidative stress and cell death [69]. Genetic factors play a crucial role in PD, with mutations in several genes associated with the disease [70]. *Alpha-synuclein* gene mutations are known to cause familial forms of PD, while mutations in the *leucine-rich repeat kinase 2 (LRRK2)* gene and *glucocerebrosidase gene (GBA)* are linked to both familial and sporadic forms of the disease [71]. These genetic mutations contribute to the development of Lewy body pathology and influence disease progression. However, even among individuals carrying the same genetic mutations, there is significant variability in disease onset and progression, highlighting the complexity of PD etiology and the interplay between genetic and environmental factors [72]. In addition to the loss of dopaminergic neurons, PD affects non-dopaminergic neurons, which contributes to a range of non-motor symptoms. These symptoms can include cognitive impairments, mood disorders, and autonomic dysfunctions such as changes in blood pressure, gastrointestinal issues, and urinary problems [73]. The exact mechanisms by which non-dopaminergic neurons are affected in PD remain unclear, but they are thought to involve the widespread spread of alpha-synuclein aggregates and disruption of various neural networks. Understanding the diverse pathological processes involved in PD is crucial for developing effective neuroprotective strategies and targeted therapies. Research continues to focus on identifying new therapeutic targets, improving disease-modifying treatments, and addressing both motor and non-motor symptoms to better manage and potentially slow the progression of PD.

## 6. Treatment Options for Cognitive Frailty, AD, and PD

No specific treatments exist for cognitive aging, as it is considered a natural aspect of the aging process. However, individuals with cognitive frailty who experience significant disruptions in daily life are often prescribed medications used for AD or PD, even without a formal diagnosis, due to shared clinical symptoms [74,75]. In AD, treatment options approved by the FDA primarily manage symptoms rather than offer a cure. Cholinesterase inhibitors, such as donepezil, rivastigmine, and galantamine, enhance acetylcholine levels to improve early-stage cognitive function [76]. Memantine, an NMDA receptor antagonist, prevents excitotoxicity caused by overactivated glutamate receptors, thereby mitigating cognitive decline in moderate-to-severe AD [77].

For PD, the most widely prescribed therapy is levodopa/carbidopa, which replenishes dopamine levels and alleviates motor symptoms [78]. Complementary treatments include dopamine agonists (e.g., pramipexole, ropinirole) to stimulate dopamine receptors and MAO-B inhibitors (e.g., selegiline, rasagiline) to prevent dopamine degradation. Anticholinergics, such as trihexyphenidyl and benztropine, reduce tremors and stiffness by inhibiting acetylcholine, while COMT inhibitors like entacapone extend levodopa’s efficacy [79]. Additional treatments include amantadine, which addresses dyskinesia through dopaminergic and anticholinergic effects, and neuroprotective agents like coenzyme Q10 and creatine, which shield neurons from oxidative damage [80]. Innovative strategies in PD management include gene therapy to boost dopamine production, stem cell therapy to replace damaged neurons, and alpha-synuclein inhibitors targeting protein aggregation, a hallmark of PD pathology [81].

Beyond pharmacological treatments, emerging therapies focus on a multimodal approach integrating both pharmacological and non-pharmacological interventions. Sodium thiosulfate offers a novel multi-targeted intervention for late-onset AD, addressing neurodegeneration on multiple levels [82]. Aerobic exercise induces neuroprotective myokines like irisin, enhancing cognitive resilience [83], while traditional Chinese medicine, such as Naofucong, targets insulin-degrading pathways to mitigate diabetic cognitive dysfunction [84]. Neuromodulation techniques, including transcranial magnetic stimulation (TMS) and neurofeedback, demonstrate efficacy in cognitive rehabilitation, particularly in neurodegenerative conditions [85].

Despite advancements, replacing lost neurons using pluripotent or neural stem cells remains a promising but underdeveloped approach for curing AD and PD, with concerns over safety, long-term efficacy, and high costs limiting widespread adoption [86]. Consequently, MSCs and their exosomes are being explored as a more feasible alternative due to their potential for neuroprotection, immunomodulation, and reparative effects. Together, these strategies underscore the importance of a comprehensive, evidence-based approach to addressing cognitive impairment, enhancing the quality of life for individuals affected by cognitive decline, AD, or PD [87].

## 7. Multipotent Mesenchymal Stromal Cell (MSC)

MSCs, first identified in 1995, are unique cells present in bone marrow and the periosteum, the bone covering [88]. MSCs secrete enzymes like superoxide dismutase (SOD) and catalase, provide anti-apoptotic benefits, and release growth factors like brain-derived neurotrophic factor (BDNF) and glial cell line-derived neurotrophic factor (GDNF), promoting neurogenesis through the activation of neural progenitor cells [89,90]. In addition, MSCs demonstrate immunomodulatory effects by stopping inflammatory microglia activation and encouraging the activation of anti-inflammatory microglia, ultimately halting additional tissue damage caused by chronic neuroinflammation [91]. Additionally, MSCs have a strong ability to replicate, enabling significant division and multiplication, making them valuable for cellular treatments [92].

Notably, MSCs possess immunomodulatory characteristics, allowing them to control immune reactions and evade immune detection, which is an essential benefit in regenerative medicine, where immune rejection is a concern [93]. Specifically, MSCs express major histocompatibility complex (MHC) I but do not have MHC II, leading to T cell deactivation and immune inhibition characteristics. MSCs also hinder the development and amount of CD80 and CD86 in dendritic cells and influence the growth and specialization of human B cells [94]. Moreover, MSCs release different chemokines, cytokines, and extracellular matrix (ECM) proteins that play a role in hematopoiesis, angiogenesis, leukocyte migration, immune functions, and inflammatory reactions. These characteristics facilitate the possibility of allogeneic MSC transplantation without requiring immunosuppression [95]. According to the International Society for Cellular Therapy (ISCT), MSCs are characterized by expressing stem markers CD73 and CD105, and not expressing hematopoietic markers CD14, CD34, and CD45. In addition, they should adhere well to plastic surfaces, have a similar appearance to fibroblasts, and retain their properties during extended periods of culture [96]. Furthermore, MSCs need to show potential for differentiation into osteogenic, adipogenic, and chondrogenic lineages. Even with these standards, MSC populations are still diverse, resulting in the term MSCs being employed for both MSC and multipotent MSC populations [97].

MSCs can be sourced from bone marrow, adipose tissue, the umbilical cord, menstrual blood, placental tissue, and amniotic fluid [98]. Bone marrow MSCs offer well-characterized cells with extensive clinical experience, but harvesting is invasive and cell quality decreases with donor age [99]. Adipose-derived MSCs are easily accessible and abundant, with minimal donor site morbidity, though they may have lower neurogenic potential [100]. Meanwhile, umbilical cord MSCs are a young cell source with high proliferation and neurogenic potential, but availability is limited and allogeneic use may require immunosuppression [101]. Menstrual blood MSCs are non-invasively collected with high neurogenic potential, though long-term stability requires more research [102]. Placental MSCs offer large cell numbers and immunomodulatory properties but are only available at birth and may raise ethical concerns [103]. Amniotic fluid MSCs are early developmental stage cells with high plasticity but have limited availability and require an invasive collection procedure [104]. One of the most fascinating qualities of MSCs is their ability to promote tissue repair by secreting factors that enhance healing and regeneration [99]. In regenerative medicine, the healing properties of MSCs primarily operate through paracrine factors, which are molecules released by the cells that impact nearby cells, regulating actions such as cell growth, movement, and specialization, ultimately aiding in tissue healing and renewal [94].

### 7.1. Research in MSC Therapy for AD

MSC transplantation is emerging as a promising therapeutic approach for AD, characterized by multifaceted effects that vary according to the disease stage [105]. In preclinical studies, MSCs have exhibited significant therapeutic potential, particularly in enhancing cellular functions associated with neuronal health and cognition [106]. Notably, MSCs boost telomerase activity and reduce tau phosphorylation, which collectively support the recovery of hippocampal neuronal structure and enhance brain glucose metabolism, which are crucial factors in mitigating cognitive decline in AD [107]. Specifically, in the early and mid-stages of AD, MSCs have been shown to inhibit the generation of Aβ. This inhibition is accompanied by a promotion of Aβ clearance, alteration in amyloid precursor protein (APP) processing, and a decrease in tau phosphorylation [108]. Moreover, MSCs enhance proteasomal activity, facilitating the breakdown of accumulated misfolded proteins, which is essential for maintaining neuronal health [99]. Meanwhile, in the later stages of AD, MSCs exert protective effects by reprogramming microglial cells, the primary immune cells in the central nervous system (CNS), transitioning from a pro-inflammatory state to an anti-inflammatory state [109]. This shift is critical, as neuroinflammation is a common feature of AD and contributes to neuronal damage. By reducing reactive microglia, MSCs help maintain neuronal integrity and support brain health. Experimental studies, including those employing the Morris water maze test, demonstrate that MSC transplantation significantly improves cognitive function [110]. Beneficial outcomes include reduced escape latency, increased platform crossings, and longer durations spent in target quadrants, indicating enhanced spatial learning and memory. These cognitive benefits are attributed to MSCs’ ability to create a neuroprotective environment, which modulates neuroinflammation, decreases oxidative stress, and reduces Aβ deposition [111].

The role of MSCs in enhancing synaptic plasticity in AD is complex, encompassing neurogenesis, synaptogenesis, and the modulation of the neuroinflammatory environment. MSCs, particularly those derived from umbilical cord and adipose tissue, have demonstrated significant potential in improving cognitive functions and synaptic health in various AD models [112]. MSCs promote neurogenesis, which is essential for synaptic plasticity. Research shows that MSC transplantation results in increased expression of synaptogenic markers like synaptophysin and neurogenic markers such as GAP-43, thereby enhancing synaptic function [113]. Additionally, MSCs secrete neurotrophic factors that stimulate local neural stem cells, encouraging the generation of new neurons and the formation of synaptic connections [114].

MSCs possess immunomodulatory properties that help reduce neuroinflammation commonly associated with AD. They activate microglial cells, facilitating the clearance of Aβ aggregates, which are characteristic of AD pathology [115]. This interaction between MSCs and the immune system not only diminishes inflammation but also fosters an environment conducive to neurogenesis and synaptic repair [114,115]. By releasing neuroprotective factors and cytokines, MSCs foster a supportive environment for neuronal connectivity and synapse formation [116].

Research indicates that MSCs promote mitochondrial function and reduce cytotoxicity, crucial for maintaining cellular health in the AD-affected brain. This mitochondrial support is vital for energy production and overall neuronal function. Research has demonstrated that transplanting mitochondria from MSCs has shown promising neuroprotective effects in cellular models of AD, significantly improving cell viability and reducing oxidative stress [117,118]. This innovative approach addresses mitochondrial dysfunction induced by Aβ aggregates, a key pathological feature of AD. By restoring healthy mitochondrial function in affected neurons, mitochondrial transplantation provides a direct mechanism to enhance neuronal resilience and functionality [118]. The ability of MSC-derived mitochondria to rejuvenate damaged cells underscores the potential of this strategy in therapeutic applications for neurodegenerative disorders. As mitochondrial dysfunction is a central aspect of AD pathology, optimizing mitochondrial transplantation techniques could pave the way for novel treatments aimed at ameliorating cognitive decline and promoting neuronal survival in AD [116,117]. Future studies should explore the mechanisms by which MSC-derived mitochondria exert their protective effects and evaluate the efficacy of this approach in vivo, potentially leading to new avenues for AD therapy.

Current reviews suggest that MSCs could provide a disease-modifying effect by targeting multiple pathways involved in AD pathology rather than focusing on a single pathological hallmark, such as amyloid plaques or tau tangles [118]. The potential of stem cell-based therapies, including MSCs, is recognized as a frontier for innovative treatments for AD. Despite significant advancements in preclinical models, the translation of these findings into clinical practice presents challenges. The complexity of AD pathology, variability among patients, and regulatory hurdles complicate the implementation of MSC therapies whereby there is a need to explore various MSC sources, including umbilical cord-derived and adipose tissue-derived MSCs, to determine their functional capabilities and effectiveness in vivo. Additionally, refining delivery methods (e.g., intravenous vs. intrathecal administration) will be crucial for maximizing therapeutic efficacy [119,120]. On the other hand, currently, there is no standardized dosage for MSC therapy in AD. Studies have reported MSC doses ranging from 10^4^ to 10^7^ cells per kilogram in preclinical models, with early clinical trials testing doses from 1 million to 100 million cells per patient [121,122]. This variability arises from differences in trial designs, administration routes, and patient characteristics. Identifying the optimal dose is vital for ensuring safety and efficacy. Future studies should delve deeper into the precise mechanisms by which MSCs exert their effects on AD pathology. Understanding these mechanisms can inform the development of targeted, personalized MSC-based therapies, offering new hope for patients and their families. While most studies report that MSC therapy is generally safe, with only minor side effects such as transient fever, the optimal dosing for AD remains undetermined [123]. The safety profile of MSC therapy in clinical settings has been encouraging, with no significant adverse effects reported, aside from mild, transient reactions [124]. However, experts emphasize the need for larger, more rigorous clinical trials to conclusively establish the long-term safety and efficacy of MSC therapies for AD.

### 7.2. Research in MSC Therapy for PD

MSCs are emerging as a promising therapeutic option for PD due to their multifaceted mechanisms of action and potential to address the underlying pathophysiology of the disease [125]. MSCs possess the remarkable capacity to differentiate into dopamine-producing neurons, a critical function for addressing the loss of neurons in PD. The degeneration of dopaminergic neurons in the substantia nigra is a defining hallmark of PD, leading to reduced dopamine levels and the progression of motor symptoms. MSCs have demonstrated the ability to adopt neuronal characteristics under specific conditions, providing a potential direct mechanism for neuronal replacement. This differentiation process could restore dopamine levels, offering a therapeutic approach to alleviate motor symptoms associated with the disease [126]. MSCs secrete a variety of neurotrophic factors, such as brain-derived neurotrophic factor (BDNF) and glial cell line-derived neurotrophic factor (GDNF). These factors play essential roles in supporting the survival, function, and regeneration of existing neurons [127]. For instance, BDNF is known to promote neuronal survival and synaptic plasticity, while GDNF specifically supports dopaminergic neuron health. This secretion contributes to an overall neuroprotective environment in the brain, enhancing the resilience of neural circuits impacted by PD [128]. The immunomodulatory properties of MSCs play a crucial role in reducing neuroinflammation, which is a key contributor to PD progression. By modulating the immune system, MSCs promote the recovery and preservation of neural tissue across various neurological disorders, including PD [129]. Their therapy for PD focuses on replacing or repairing damaged brain cells and restoring normal dopamine-producing brain cell function, alleviating motor symptoms. Studies showed that when injected intravenously, MSCs utilize their “homing” mechanism to locate damaged and inflammatory sites within the brain. This targeted approach allows them to effectively modulate the immune response and reduce inflammation, including neuroinflammation, thereby potentially slowing PD progression [130]. Despite these encouraging findings, the clinical translation of MSC-based therapies for PD still faces several challenges. Many studies have been limited by small sample sizes, short follow-up periods, and variability in the types and sources of stem cells used, leading to inconclusive results regarding their long-term efficacy and safety [131]. Additionally, optimizing the therapeutic protocols, including determining the most effective stem cell type, the appropriate cell modifications, the optimal number of cells to be transplanted, and the best delivery methods, remains a critical area of ongoing research [132,133]. Addressing these challenges will be essential for advancing MSC therapy from the laboratory to clinical practice, ensuring that it can provide a reliable and effective treatment option for patients with PD.

## 8. Exosomes

Exosomes are nanosized membrane microvesicles, typically ranging from 30 to 150 nanometers (nm) in diameter [134]. Their small size allows them to effectively traverse biological barriers and interact with target cells, making them important mediators of intercellular communication [135]. These nanosized extracellular vesicles are released by different cell types, including stem cells. These exosomes act as natural carriers, delivering various biomolecules such as proteins, lipids, and nucleic acids to target cells [136]. Exosomes derived from stem cells, including neural stem cells (NSCs), MSCs, and embryonic stem cells (ESCs), have garnered significant attention for their potential therapeutic applications in neurological disorders [137]. The therapeutic potential of exosomes in neurological conditions is attributed to their ability to cross the blood–brain barrier, target specific cell types, and deliver their cargo of biomolecules to degenerated or injured sites [138]. The specific mechanisms and effects observed in each neurological condition may vary depending on the exosome source, cargo, and the underlying pathophysiology [139]. The intricate mechanisms involved in exosome biogenesis, including both ESCRT-dependent and ESCRT-independent pathways, highlight the complexity of their formation and cargo loading [140]. Understanding these mechanisms is essential for harnessing exosomes for therapeutic purposes effectively. The potential of exosome therapy in neurological disorders is promising, whereby the broader application of MSCs in treating neurodegenerative diseases beyond PD has also been explored, showing their potential in conditions like AD and multiple sclerosis via promoting neurorestoration and cognitive function through various mechanisms, including neurogenesis, anti-inflammatory effects, and modulation of synaptic function [140,141].

### 8.1. Research in Exosome Therapy for AD

Extracellular vesicles (EVs), including exosomes derived from MSCs, have shown promising therapeutic effects in AD models [142,143,144]. MSC-derived EVs, administered via various routes such as intravenous (IV) or lateral ventricle injection, have demonstrated benefits including reduced Aβ deposition, improved cognitive function, increased neuronal viability, and modulation of inflammatory and apoptotic responses [145]. The therapeutic mechanisms involve the activation of neuroprotective pathways, expression of beneficial microRNAs, and reduction of pro-inflammatory factors. A study also found that MSC-exosomes could inhibit reactive astrocytes and activated microglia, and modulate microRNA levels affecting histone deacetylase 4 (HDAC4), which is implicated in AD [146]. These findings support further research into optimizing MSC-exosome therapy for AD and understanding its underlying mechanisms. In another study, exosomes conjugated with a central nervous system-specific rabies viral glycoprotein (RVG) peptide (MSC-RVG-Exo) demonstrated a significant reduction in amyloid-beta (Aβ) plaque deposition and astrocyte activation, and improved cognitive function. Additionally, MSC-RVG-Exo treatment significantly reduced pro-inflammatory cytokines (TNF-α, IL-β, IL-6) and increased anti-inflammatory cytokines (IL-10, IL-4, IL-13), demonstrating an effective method for enhancing brain-targeted therapy in AD [147].

### 8.2. Research in Exosome Therapy for PD

**Figure 1:**
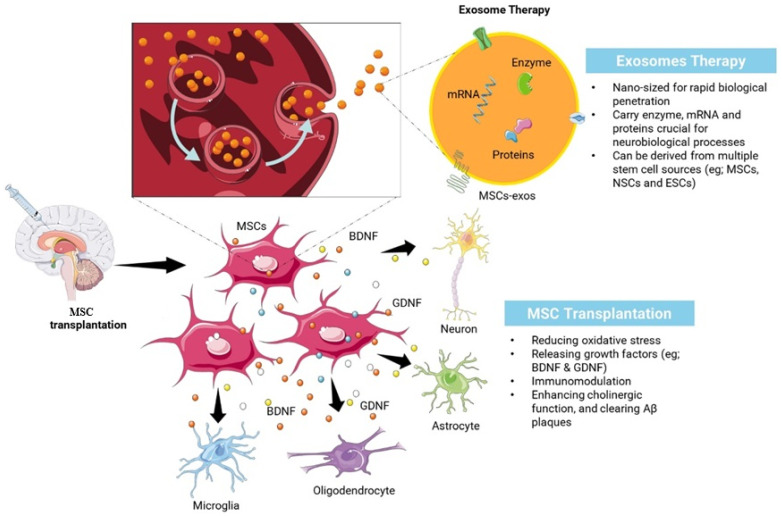
The cellular-level workings of MSC transplantation and exosome therapy.

Studies have shown that MSC-derived exosomes can effectively cross the blood–brain barrier, playing a critical role in neuroprotection in PD models. These exosomes induce autophagy, inhibit apoptosis, and promote cell proliferation, offering multifaceted benefits in PD therapy [148,149]. In vivo experiments further highlight their ability to reduce dopaminergic neuron loss, upregulate dopamine levels in the striatum, and alleviate PD symptoms [149]. Although the precise mechanisms are not fully understood, MSC-derived exosomes present a promising alternative to traditional stem cell therapies, potentially overcoming challenges like uncontrollable differentiation and providing a more controlled therapeutic approach [149,150]. Another study found that exosomes produced by MSCs could keep human brain microvascular endothelial cells (HBMECs) in a transcriptionally active state, which may promote angiogenesis—a process beneficial for neuroprotection and tissue repair in PD models [151]. This suggests that MSC-derived exosomes not only offer neuroprotective benefits but may also enhance the repair of damaged blood vessels in the brain. Studies have demonstrated that exosomes containing catalase, an enzyme with neuroprotective properties, can reach neurons and exert beneficial effects in PD models [152]. These exosomes were also found to facilitate therapeutic benefits by crossing the blood–brain barrier and delivering neuroprotective agents like antioxidants, catalase, and GDNF [153]. Despite these encouraging findings, several challenges remain, such as low yields of exosomes, and difficulties in their isolation and purification pose significant obstacles to their widespread clinical use [154]. Addressing these challenges, alongside rigorous clinical trials, will be essential for translating the therapeutic potential of MSC-derived exosomes into practical and effective treatments for PD and other neurodegenerative disorders. Table 2 provides a comprehensive comparison between MSCs and exosomes in terms of their therapeutic potential, mechanisms, safety, and development challenges. Figure 1 highlights the cellular-level workings of both therapies: MSC transplantation emphasizes direct interaction with brain cells, secretion of growth factors, and regenerative effects, while exosome therapy focuses on a cell-free strategy that leverages nanosized vesicles to deliver therapeutic molecules efficiently.

### 8.3. Practical Aspects of MSC vs. Exosome Therapies

Both MSC therapy and exosome therapy show potential in regenerative medicine, each offering distinct benefits and drawbacks. MSCs have a reputation for their ability to regenerate tissue, influence the immune system, and navigate towards areas of damage or inflammation, which allows for their versatility in treating various ailments such as AD and PD [155]. They have the ability to release many bioactive molecules that aid in tissue healing and regulate the immune system [156]. Nevertheless, there are also substantial obstacles associated with MSC therapy. Variability among cell populations may result in inconsistent therapeutic results, posing a potential risk of tumor formation [157]. Furthermore, the clinical application of these cells is complicated by logistical challenges like harvesting, expanding, maintaining, and potential immune rejection [158].

**Figure 2:**
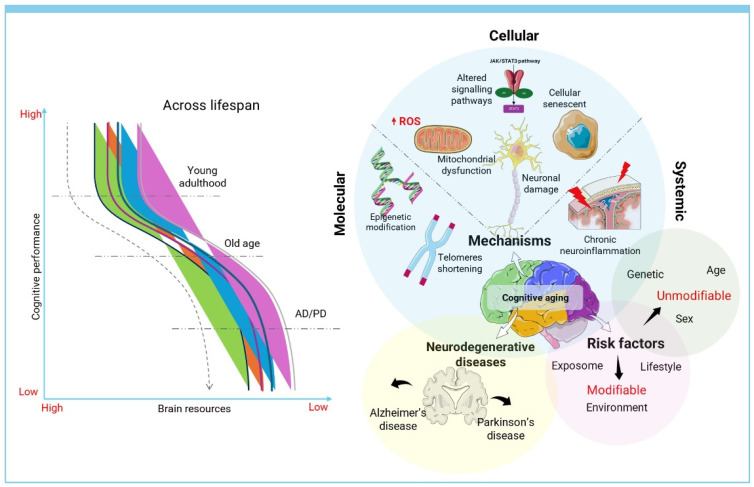
Comprehensive overview of cognitive aging: mechanisms, risk factors, and neurodegenerative disease pathways.

On the other hand, exosome therapy provides a cell-free option that circumvents certain challenges linked to live cell transplants [159]. Exosomes are small vesicles that transport proteins, lipids, and nucleic acids and have the ability to pass through biological barriers, which allows them to efficiently deliver therapeutic substances to specific cells. Due to their compact size and stability, making them easier for standardization and manufacturing, they also have low immunogenicity, decreasing the chances of immune rejection [160]. Exosomes have the practical benefit of being able to be stored for extended periods without a notable decrease in function when compared to live cells. Yet, exosome treatment comes with drawbacks as well. Exosomes have a lower homing efficiency than MSCs, potentially impacting their therapeutic effectiveness [161]. Identifying the best dosing and administration methods for exosomes poses difficulties, and the isolation and characterization procedures remain intricate and not fully uniformed [162]. Moreover, there is still a lack of complete understanding regarding the specific ways in which exosomes produce their therapeutic benefits, highlighting the need for additional research. Figure 2 provides a comprehensive overview of cognitive aging, combining cognitive performance trends across the lifespan with biological mechanisms and risk factors.

## 9. Discussion

Cognitive aging, a natural and multifaceted process, is characterized by gradual declines in various cognitive functions such as memory, attention, and executive function (7 and 11). While these changes can coexist with periods of stability or even improvement in certain cognitive domains, significant cognitive impairment that disrupts daily life often marks the transition from normal aging to pathological cognitive decline (8 and 19). This transition may manifest as MCI and, in some cases, progress to more severe forms such as AD or PD [46]. Both AD and PD represent the extreme end of the cognitive aging spectrum, characterized by clinically significant deficits that severely affect daily functioning and quality of life [38]. In AD, the pathological hallmark includes the accumulation of amyloid-beta plaques and tau tangles, which lead to neuroinflammation, synaptic loss, and neuronal death, starting in memory-related areas such as the hippocampus. As the disease progresses, it extends to other cortical areas, resulting in profound impairments in multiple cognitive functions, including memory, language, and executive function [52,53,54,55]. PD, on the other hand, primarily involves the degeneration of dopaminergic neurons in the substantia nigra, leading to motor deficits. However, cognitive deficits also emerge as PD progresses, especially involving executive function, attention, and memory [13,14].

Given the increasing life expectancy worldwide, the prevalence of cognitive aging and neurodegenerative diseases like AD and PD is expected to rise, creating an urgent need for effective treatments that go beyond symptomatic management. Current pharmacological treatments, such as acetylcholinesterase inhibitors in AD [77] and levodopa in PD [79], provide only limited symptomatic relief, with no cure available. These challenges have spurred interest in novel therapeutic approaches, particularly those that aim to address the underlying causes of cognitive decline rather than simply alleviating symptoms. Among these emerging therapies, MSCs and their derived exosomes have garnered significant attention due to their regenerative, neuroprotective, and immunomodulatory properties [99,140].

MSCs, which can be sourced from various tissues such as bone marrow, adipose tissue, and umbilical cord tissue, have demonstrated the potential to secrete a wide array of bioactive molecules, including growth factors, cytokines, and EVs. These secreted factors are believed to exert several beneficial effects, including promoting neurogenesis, protecting neurons from oxidative stress, and modulating immune responses [87,88]. In neurodegenerative diseases such as AD and PD, where inflammation, oxidative stress, and neurogenesis deficits contribute to disease progression, MSCs have shown promise in slowing or even reversing cognitive decline [69]. Through their secreted factors, MSCs can provide neurotrophic support and modulate the neuroinflammatory environment, potentially addressing the underlying pathophysiology of these diseases [163].

Exosomes, which are nanosized extracellular vesicles secreted by MSCs, represent a particularly exciting avenue for therapeutic development [140]. These vesicles contain a variety of bioactive molecules, including proteins, lipids, and RNA, which can exert neuroprotective effects and facilitate the clearance of amyloid-beta plaques in AD models [164]. Exosomes have the added advantage of being able to cross the blood–brain barrier (BBB), which remains a significant challenge in the treatment of neurodegenerative diseases [165]. The ability of exosomes to efficiently deliver therapeutic payloads to the brain, while minimizing immune rejection, makes them an attractive method for targeted therapy in AD, PD, and other cognitive disorders. Additionally, exosomes have been shown to promote autophagy, inhibit apoptosis, and support neuronal survival, making them an ideal candidate for treating both AD and PD, where these processes are often disrupted [149,166].

Despite the promising potential of MSCs and exosomes, several challenges remain before these therapies can be fully integrated into clinical practice. The efficiency of exosome isolation, their stability during storage and transport, and the ability to enhance their targeting to specific regions of the brain are key areas of ongoing research [167]. Moreover, while MSC-based therapies are generally considered safe, their long-term effects and optimal dosages have not yet been fully established, and clinical trials are necessary to determine their efficacy in humans [168]. Furthermore, the integration of MSCs and exosomes into clinical settings requires the development of standardized protocols to ensure reproducibility, safety, and efficacy [99,140]. However, further research is essential, since as research into neurodegenerative diseases progresses, precision medicine is increasingly shaping personalized healthcare strategies by identifying specific genetic mutations and environmental factors that influence conditions like AD and PD. Early detection through biomarker analysis, such as amyloid-beta and tau proteins in neuroimaging or cerebrospinal fluid tests, offers opportunities for preventive interventions before symptoms emerge [169]. Lifestyle factors, including diet, exercise, and cognitive training, play a crucial role in modulating disease risk, and digital health technologies combined with global research efforts will further enhance early interventions [22]. The integration of emerging therapies, such as stem cell and exosome-based treatments, with lifestyle changes provides a comprehensive approach to addressing cognitive frailty. However, challenges in delivery and blood–brain barrier (BBB) penetration remain, requiring innovative strategies like ligand modifications, osmotic disruption, and alternative delivery routes [170]. Additionally, the large-scale production of extracellular vesicles (EVs) using optimized bioreactor systems will be vital for advancing clinical applications, ensuring high-yield, standardized, therapeutic-grade EVs for neuroregenerative therapies [171].

## 10. Conclusions

Cognitive impairment, particularly in AD and PD, represents a growing challenge in the context of global aging. This review has explored the interconnected yet distinct mechanisms of cognitive aging and impairment, highlighting their relevance to neurodegenerative diseases. Cognitive aging is a natural process marked by gradual declines in memory, attention, and executive function, influenced by genetic and lifestyle factors. In contrast, cognitive impairment involves clinically significant deficits that disrupt daily life and often result from pathological conditions, including neurodegenerative diseases, cerebrovascular events, and systemic factors. AD and PD exemplify the continuum between normal cognitive aging and pathological decline, sharing mechanisms such as neuroinflammation, oxidative stress, and structural brain changes, while displaying distinct clinical trajectories. The review underscores the importance of distinguishing age-related changes from disease-specific impairments to enable timely interventions. While some cognitive functions remain stable or improve with age, pathological processes often lead to severe declines, necessitating targeted therapeutic approaches. Emerging strategies, such as MSCs and their exosome-derived therapies, hold promise for mitigating cognitive decline and enhancing quality of life. Advancing our understanding of the shared and unique aspects of AD and PD will refine treatment strategies and better support individuals at risk of cognitive decline. Promoting healthy cognitive aging alongside effective management of neurodegenerative diseases remains a critical goal, requiring continued research and innovative therapeutic development.

## Figures and Tables

**Table 1:** Comparative overview of key differences between cognitive aging and cognitive impairment.

Aspect	Cognitive Aging	Cognitive Impairment
Definition	A natural and gradual process of cognitive changes due to aging.	Clinically significant deficits in cognitive function often linked to diseases or other medical conditions.
Pathology	Non-pathological; part of normal aging.	Pathological; caused by underlying conditions such as Alzheimer’s Disease (AD), Parkinson’s Disease (PD), or systemic illnesses.
Nature of Changes	Gradual declines in processing speed, memory, and attention, with compensatory neural mechanisms.	Abrupt or progressive deficits that interfere with daily life, such as memory loss, confusion, or executive dysfunction.
Key Mechanisms	Neural plasticity, cognitive reserve, and compensatory scaffolding.	Inflammation, oxidative stress, vascular damage, neurodegeneration, or systemic dysfunctions.
Impact on Daily Function	Typically mild and does not interfere significantly with daily life.	Impairs daily functioning and quality of life.
Examples of Influences	Education, mental engagement, physical activity, and overall health.	Conditions like AD, PD, type 2 diabetes, obesity, chemotherapy, COVID-19, and schizophrenia.
Interventions	Lifestyle modifications such as mental and physical exercises, social engagement, and nutrition.	Disease-specific treatments, neuroprotective strategies, and rehabilitative interventions like dual tasks or probiotics.
Biomarkers	Lack of specific pathological biomarkers; relies on general markers of aging.	May include epigenetic markers, NMDAR hypofunction, and systemic inflammatory markers.

**Table 2:** Comparative summary of MSCs and exosomes.

Aspect	Mesenchymal Stromal Cells (MSCs)	Exosomes
Physical Characteristics	Living cells Require specific culture conditions Need to maintain cell viability Express specific markers (CD73, CD105) Fibroblast-like appearance	Nanosized membrane microvesicles 30−150 nanometers in diameter Cell-free vesicles More stable than whole cells Contain proteins, lipids, and nucleic acids
Source Options	Bone marrow Adipose tissue Umbilical cord Menstrual blood Placental tissue Amniotic fluid	Can be derived from various stem cells including: - Neural stem cells (NSCs) - MSCs - Embryonic stem cells (ESCs)
Delivery and Distribution	May have limited ability to cross biological barriers Requires consideration of cell survival Multiple administration routes (IV, intrathecal) Cell size may limit distribution	Effectively traverse biological barriers Can cross blood–brain barrier Better distribution due to small size Can be targeted to specific cell types
Therapeutic Mechanisms	Direct cell replacement Secretion of therapeutic factors Immunomodulation Anti-inflammatory effects Paracrine signaling Direct cell-to-cell contact	Delivery of bioactive molecules Intercellular communication Transfer of proteins and nucleic acids Modulation of recipient cell behavior No direct cell replacement
Safety Considerations	Risk of uncontrolled differentiation Potential immune responses Need for immunosuppression in some cases Cell survival challenges Generally safe with minor side effects	Lower risk profile No risk of uncontrolled differentiation More controlled therapeutic approach May have better safety profile
Production Challenges	Requires complex culture conditions Cell quality varies with donor age Limited scalability Storage and transport challenges Need to maintain cell viability	Low yield in production Difficulties in isolation and purification Challenges in standardization Storage may be easier than cells
Disease-Specific Effects	Alzheimer’s Disease: Reduces tau phosphorylation Enhances proteasomal activity Promotes Aβ clearance Reprograms microglial cells Improves cognitive function Parkinson’s Disease: Can differentiate into dopaminergic neurons Direct cell replacement potential Secretes neurotrophic factors Modulates immune response Reduces neuroinflammation	Alzheimer’s Disease: Reduces Aβ deposition Improves cognitive function Modulates inflammatory responses Increases neuronal viability Can be enhanced with targeting peptides (e.g., RVG) Parkinson’s Disease: Induces autophagy Inhibits apoptosis Promotes cell proliferation Reduces dopaminergic neuron loss Can deliver specific therapeutic agents (e.g., catalase)
Current Limitations	Complex regulatory requirements Variability in therapeutic effects Storage and transport challenges Cost of production Need for standardized protocols	Low production yield Purification challenges Limited understanding of mechanisms Need for standardization Scale-up difficulties
Future Development Needs	Standardization of protocols Optimization of delivery methods Better understanding of mechanisms Larger clinical trials Cost reduction strategies	Improved isolation methods Better production yields Enhanced targeting strategies Standardized characterization More clinical evidence

## Data Availability

There are no data to support the findings of this review.
